# The Relationship Between Fatigue, Pain Interference, Pain-Related Distress, and Avoidance in Pediatric Hypermobile Ehlers–Danlos Syndrome

**DOI:** 10.3390/children12020170

**Published:** 2025-01-29

**Authors:** Olivia E. Sokol, Emma E. Biggs, Ardin S. Berger, Laura E. Simons, Rashmi P. Bhandari

**Affiliations:** 1 Department of Anesthesiology, Perioperative and Pain Medicine, School of Medicine, Stanford University, Stanford, CA 94305, USA; eebiggs@stanford.edu (E.E.B.); ardinberger@edscomfortclinic.com (A.S.B.); lesimons@stanford.edu (L.E.S.); rbhandar@stanford.edu (R.P.B.); 2 Comfort Clinic, Berkeley, CA 94705, USA

**Keywords:** hypermobile Ehlers–Danlos syndrome, pediatric pain, fatigue, hypermobility, pain-related distress

## Abstract

Background/Objectives: Fatigue is a known predictor of disability and reduced quality of life in youth with hypermobility and chronic pain in general. Given the added relationship between chronic fatigue and connective tissue disorders, including hypermobile Ehlers–Danlos Syndrome (hEDS), this study aims to investigate the comparative role of fatigue on important predictors of outcomes for youth with and without hEDS who have chronic pain. Methods: In this retrospective study, pediatric patients with chronic pain diagnosed with hEDS (*n* = 100) were compared to an age- and sex-matched group of youth with chronic pain without diagnosed hypermobility (*n* = 100). Participants completed measures of pain-related distress (PCS-C), avoidance (FOPQ-A), and pediatric PROMIS measures for fatigue, anxiety, and pain interference. Data were analyzed using chi-square tests, *t*-tests, and ANCOVAs in RStudio. Results: Fatigue scores were higher and clinically elevated fatigue was more prevalent in those with hEDS than in matched chronic pain peers. Fatigue was significantly positively related to pain interference, avoidance, and pain-related distress in youth with and without hEDS. Conclusions: The current study supports the need for multidisciplinary treatment and rehabilitation for pediatric chronic pain and hypermobility and suggests that fatigue may be an important factor to consider when treating youth with hypermobility.

## 1. Introduction

Hypermobile EDS (hEDS) is the most common subtype of Ehlers–Danlos syndrome (EDS) [1]. While studies offering an accurate estimation of prevalence for hEDS are lacking in the current literature [1], the prevalence is presumed to be, at minimum, 1 in 5000 [1,2]. EDS, including hEDS, has historically been considered a rare disease; however, emerging research suggests that the prevalence of EDS is underestimated and challenges the assertion that EDS is a rare disease [2,3]. Based on data from a large population survey of adults [4], an estimated 10 million individuals in the US and 225 million individuals worldwide are impacted by hEDS [1]. Notably, hEDS is the only remaining subtype of Ehlers–Danlos syndrome lacking a genetic marker, and it is thought to display an autosomal dominant inheritance pattern with incomplete penetrance and variable expressivity [5], meaning that some individuals who carry the relevant genes will express symptoms of hEDS while others will not, and symptoms range between individuals. Youth with hypermobility experience greater fatigue, pain, and disability compared with normative data [6] and non-pain peers [7]. Among youth with chronic musculoskeletal pain, the prevalence of generalized joint hypermobility is 35–48% [8]. About a third of children aged 0–10 with hEDS report chronic fatigue, back pain, arthralgias, and myalgias, which increases to two-thirds of patients between the ages of 11 and 20 [5,9]. While youth with hEDS experience unique symptoms compared to those with other chronic pain conditions [10], there is a lack of research comparing youth with hEDS to those with other types of chronic pain; such research would help clinicians optimize tailored treatment for those with hEDS, as this multisystemic condition tends to be related to higher levels of disability [11] and greater trajectory towards a decreased quality of life [12].

Fatigue is thought to be an especially difficult and important component of living with hypermobility-related disorders [10,13,14]. Fatigue can be conceptualized as a multidimensional construct with cognitive, physical, motivational and emotional dimensions [15]. While fatigue is impacted by sleep quality, fatigue and daytime sleepiness are independent phenomena [16]. Prior research indicates that fatigue is associated with hypermobility via biological mechanisms such as autonomic dysfunction and altered interoception [17]. Autonomic dysregulation directly affects the sleep–wake cycle and is, in turn, directly affected by abnormal sleep patterns [18]. Fatigue has also been linked to hEDS by common comorbidities including symptoms of mast cell activation and postural orthostatic tachycardia syndrome (POTS) [5,19]. Additional possible contributors of chronic fatigue in people with hEDS include pain, physical deconditioning, and muscle weakness [5,20]. As hEDS involves autonomic dysfunction and muscular hypotonicity, the effect of fatigue on pain interference could be more pronounced than in other chronic pain conditions through cardiac and muscular deconditioning [17]. This association is thought to be at least partially attributable to the relationship between autonomic dysregulation and heritable connective tissue disorders, with secondary increased risk for symptoms of anxiety and stress [21].

The consequence of fatigue is the negative impact on functional outcomes in individuals with hypermobility such as health-related quality of life, disability, depressive symptoms, and social functioning. In qualitative interviews, adults with hEDS describe pain, fatigue, and anxiety regarding their condition as burdens of daily living [22,23]. In adult participants with hEDS, severe fatigue, high anxiety, and pain-related distress have been shown to be associated [24]. Fatigue negatively impacts daily functioning, social functioning, and mood in adults with EDS [25], and fatigue severity was found to be predictive of functional disability in youth with hEDS [10]. From the current literature, it is unclear whether fatigue is more severe in youth with hEDS compared to peers with other chronic pain, and whether fatigue is differently associated with factors such as pain-related distress or pain interference.

Current research on fatigue and pain in youth with hEDS is in its infancy [26], and there is a need for more quantitative research on how youth with chronic pain with and without hEDS have differing experiences. Higher fatigue severity has been shown to be related to higher anxiety and depression in youth with hEDS [10], and further research is needed to examine the impact of fatigue on pain-related distress. Additional research investigating psychological factors related to pain and disability in this population is also needed [27]. Furthermore, there is a lack of quantitative research on pain-related fear and distress in youth with hEDS [8]. Overall, research in this emerging area of study is often exploratory (*n* < 50) [7,28,29], combines populations of youth with hEDS and non-hEDS hypermobility [28,30], has no comparison group [28,29], or uses non-clinical, healthy, or asymptomatic hypermobile populations as controls [7]. It is important to further study the relationship between physical and psychological symptoms in youth with hEDS [10] in order to understand how overlapping symptoms contribute to disability, and to inform and tailor interdisciplinary care for this population.

Elucidating potential modifiable mechanisms connecting fatigue with functional outcomes may aid in informing personalized patient care. Pain-related distress and pain-related fear which leads to avoidance of activities are two established predictors of poor outcomes in chronic pain. Pain-related distress, often conceptualized as pain catastrophizing, is a powerful predictor of poor outcomes, including higher pain intensity, higher functional disability, and poorer health-related quality of life, in youth with pain [31]. Pain-related fear, including avoidance, has been shown to be associated with disability and depression and to predict poor treatment response in youth with chronic pain [32,33]. Activity avoidance is a particularly salient dimension of pain-related fear, influencing disability and school functioning [33]. Notably, previous research found that fear of pain and pain avoidance were strongly related to fatigue severity in individuals with hEDS [34]. As pain intensity and anxiety have been known to impact pain interference, pain-related distress, and avoidance [35,36,37,38], these variables will be controlled in the current study analyses. The impact of fatigue on pain related distress and avoidance of activity has not been directly studied in youth experiencing chronic pain with and without hEDS.

This study is the first to compare youth with hEDS and youth with other types of chronic pain to investigate potential differences in fatigue experience in terms of prevalence, intensity, and the relationship of fatigue and pain interference as well as pain-related distress. The study first aims to underscore what is already known in the existing literature—that fatigue is severe and prevalent in youth with hEDS—and to add to that literature by comparing youth with hEDS to peers who have chronic pain without hEDS. The hypotheses for aim 1 are that (a) youth with hEDS have greater prevalence of clinically elevated fatigue, and (b) youth with hEDS have a higher mean fatigue score compared to the CP group. Secondly, the study aims to highlight that fatigue is significantly related to clinically meaningful predictors of outcomes in youth with hEDS such as pain interference, pain-related distress, and avoidance. The hypotheses for aim 2 are that fatigue is significantly related to (a) pain interference, (b) pain-related distress (measured by PCS), and (c) avoidance. Third, the study aims to understand whether there is a differential relationship between fatigue and the variables of pain interference, pain-related distress, and avoidance when comparing the hEDS and CP groups. The hypotheses for aim 3 are that there is a difference between the hEDS and CP groups in the relationship between fatigue and (a) pain interference, (b) pain-related distress, and (c) avoidance, with the relationship being stronger for the hEDS group.

## 2. Materials and Methods

### 2.1. Participants

Youths presenting to a tertiary pediatric pain clinic completed a baseline assessment prior to evaluation as part of usual care [39]. The STARR (Stanford Research Repository) database was searched for patients who completed evaluations between 1 April 2017 and 28 February 2023 and were given an explicit diagnosis of hypermobile Ehlers–Danlos Syndrome. Inclusion and exclusion criteria for the cohort pulled from STARR were manually confirmed by the authors using chart review. This resulted in a final sample of 100 youth with hEDS. A second search was then conducted to identify 100 youth without hypermobility, matched to the hEDS sample on legal sex, age, and year of evaluation.

The two study groups were the hEDS group—youth with chronic pain and diagnosed hEDS—and the CP group—youth with chronic pain and no diagnosed hypermobility. Inclusion and exclusion criteria for each group were as follows:

hEDS group—Inclusion Criteria

- Diagnosed with hypermobile Ehlers–Danlos syndrome (Q79.62, ICD-10) on or after 1 April 2017;
- Evaluated between 1 April 2017 and 28 February 2023 at the pain clinic;
- Aged 8—17 years old at evaluation.

hEDS group—Exclusion Criteria

- Non-English speakers;
- No baseline survey completed;
- No diagnosis of hEDS.

CP group—Inclusion Criteria

- Evaluated between 1 January 2017 and 28 February 2023 at the pain clinic;
- Diagnosis of chronic pain (e.g., headache, musculoskeletal or nerve pain).

CP group—Exclusion Criteria

- Non-English speakers;
- No baseline survey completed;
- Diagnosis of hEDS or HSD (Hypermobility Spectrum Disorder).

For further context, the hEDS diagnosis date of 1 April 2017 or later in the inclusion criteria for the hEDS group was selected because the diagnostic criteria for Ehlers–Danlos syndromes were updated in March of 2017, when the International EDS Consortium proposed revised clinical criteria to be clearer and more specific [40]. As hEDS is a lifelong condition that is often diagnosed with a significant delay and may not be clinically feasible to evaluate at initial visit, baseline surveys were used from patients diagnosed with hEDS at initial evaluation or at a later follow-up appointment with their pain physician.

### 2.2. Procedure

Patients and their primary caregiver completed a baseline Peds-CHOIR (Pediatric Collaborative Health Outcomes Information Registry) gathering demographic information and assessing mood and pain-related functioning prior to their initial interdisciplinary pain management evaluation. Patient and caregiver each completed their own Peds-CHOIR survey for the baseline. Pediatric measures of pain, mood, and pain-related functioning were analyzed for the study. Peds-CHOIR includes National Institutes of Health (NIH) Patient-Reported Outcomes Measurement Information System (PROMIS) domains as well as legacy measures. Surveys were deployed electronically via a secure URL link over email or in the clinic waiting room on encrypted tablets. Peds-CHOIR is accessed through a university-approved Oracle database. Study procedures were approved by the university’s institutional review board as part of a larger retrospective patient chart review study (#40564) and informed consent was not required as the information was collected as part of standard clinical care. For the hEDS group, a cohort of patients with hEDS, diagnosed after 31 March 2017, were selected using the STARR cohort search. The inclusion and exclusion criteria for the hEDS group were applied to the STARR cohort using chart review, resulting in a sample size of 100 patients included in the hEDS group. For the CP group, a cohort of patients were matched to the hEDS group by selecting youth with a matching legal sex, age, and year of evaluation for each patient in the hEDS group. Data were extracted retrospectively from Peds-CHOIR and patient medical charts.

### 2.3. Measures

#### 2.3.1. PROMIS Measures

The Patient-Reported Outcomes Measurement Information System (PROMIS) measures were developed by the NIH to provide clinicians and researchers with validated patient-reported outcome measures for domains of physical, mental, and social health, and increase comparability across studies. Measure questions use a Likert scale (1 = “never or not able to do” to 5 = “almost always or with no trouble”). This study utilized Computer Adaptive Testing versions of the PROMIS measures and item response theory [41]. Scores are based on T-score distribution with a mean of 50 points and a standard deviation of 10. PROMIS measures are normed against the general US population and multiple chronic disease populations [42], and pediatric measures are validated in children aged 8–17 years old [43].

PROMIS Pediatric Fatigue

The PROMIS pediatric fatigue item bank evaluates the youth’s experience of tiredness and exhaustion and the impact of fatigue on youth day-to-day, cognitive, emotional, and social functioning (e.g., “I was so tired it was hard for me to pay attention,” “I felt weak”) over the past 7 days. Higher scores demonstrate worse fatigue.

PROMIS Pediatric Anxiety Symptoms

The PROMIS pediatric anxiety item bank examines youth worries (e.g., “I felt like something awful might happen”) over the past 7 days. Higher scores demonstrate greater anxiety symptoms.

PROMIS Pediatric Pain Interference

The PROMIS pediatric pain interference item bank evaluates the impact of pain on youth day-to-day, cognitive, emotional, and social functioning (e.g., “I had trouble doing schoolwork when I had pain,” “It was hard for me to have fun when I had pain”) over the past 7 days. Higher scores indicate greater pain interference.

#### 2.3.2. Other Measures

Demographics

Patient age at baseline, legal sex, race, and ethnicity were extracted from medical chart.

Pain Intensity

Participants rated their average pain intensity in the week prior to their evaluation using a standardized 11-point numeric scale (0 = no pain, and 10 = worst pain possible) which has shown validity in the pediatric pain population [44].

Pain Catastrophizing Scale for Children (PCS-C)

The PCS-C, adapted from the adult PCS, assesses thoughts and feelings of pain-related distress (e.g., “When I am in pain, it’s terrible and I think it’s never going to get better”) and is validated for youth [45]. The PCS-C is a 13-item measure with a 5-point Likert scale (0 = “Not at all” to 4 = “Extremely”). Higher scores indicate greater levels of pain-related distress.

Fear of Pain Questionnaire, Child Report—Short Form

The FOPQ-SF Child assesses pain-related fears and has two subscales: Fear of Pain (e.g., “Feelings of pain are scary for me”) and Avoidance of Activities (e.g., “I avoid making plans because of my pain”). This 10-item measure is rated on a 5-point Likert-type scale (0 = “strongly disagree’” to 4 = “strongly agree”). The measure is reliable and valid in youth with chronic pain [46]. Higher scores indicated greater fear of pain and avoidance of activities.

### 2.4. Data Analysis

Analyses were conducted in RStudio (2022.12.0 + 353). *t*-tests and chi-square tests were used to compare baseline demographics (race, ethnicity) and pain intensity between groups. Data were examined visually for normality. Hypotheses were determined a priori. For Hypothesis 1a, a chi-square test was used to compare prevalence of clinically elevated fatigue between groups, and a *t*-test was used for Hypothesis 1b to compare mean fatigue scores between groups. For Hypotheses 2a–c, analyses of covariance (ANCOVA) were used to examine the relationship between fatigue and pain interference, pain-related distress, and avoidance in the hEDS group only. For Hypotheses 3a–c, ANCOVAs were used to examine interaction effects for group in the relationship between fatigue and pain interference, pain-related distress, and avoidance in both groups. Both ANCOVA models controlled for anxiety and pain intensity.

## 3. Results

### 3.1. Demographics

The sample of youth were aged 9–17 (*M* = 14.67, *SD* = 1.98). The hEDS and CP groups, matched by legal sex, age, and year of evaluation, did not differ significantly by race or ethnicity. Insufficient data were available to evaluate gender. See Table 1 for full demographics.

### 3.2. Pain Intensity and Anxiety

The hEDS and CP groups did not differ significantly in pain intensity or anxiety. See Table 1.

### 3.3. Fatigue Prevalence and Intensity: Hypothesis 1 (H1a–H1b)

**Figure 1:**
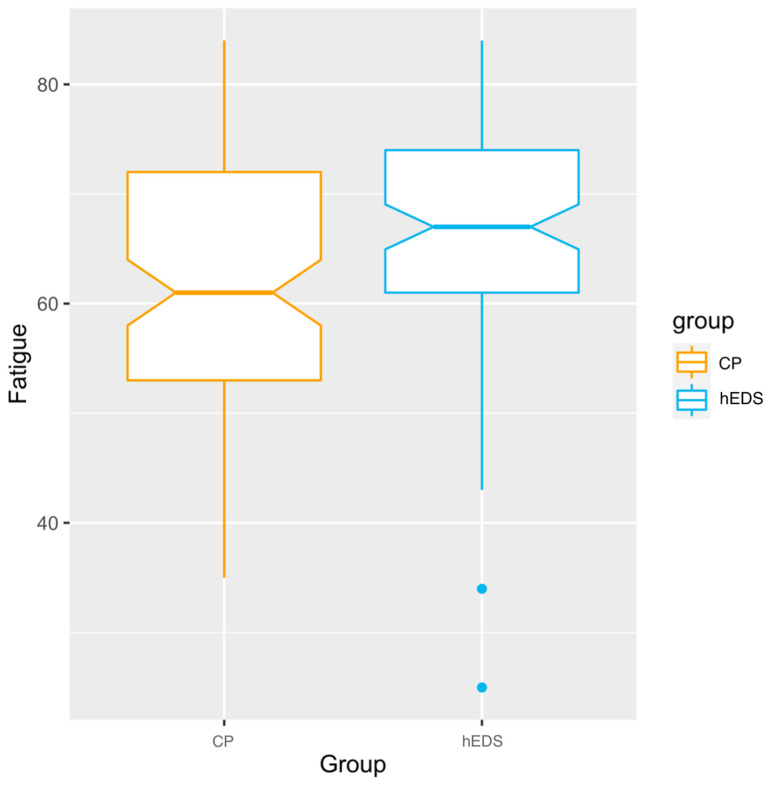
PROMIS pediatric fatigue scores in the hEDS and CP groups. Legend: hEDS = group with hypermobile Ehlers–Danlos syndrome; CP = group with chronic pain and no diagnosed hypermobility; Fatigue = PROMIS pediatric fatigue scores.

A chi-square test was used to test whether the prevalence of clinically elevated fatigue (severe fatigue, PROMIS score of 65 or greater) was higher in those with hEDS compared to matched CP peers (Hypothesis 1a; see Figure 1). As expected, the groups were significantly different (X^2^(1) = 6.32; *p* = 0.007), with a greater prevalence in the hEDS group (hEDS: 59% clinically elevated fatigue; CP: 39% clinically elevated fatigue). Furthermore, mean fatigue scores were significantly higher in those with hEDS (M_hEDS_ = 66.25) compared to matched CP peers (M_CP_ = 61.60) (H1b; t(194.15) = 2.96, *p* = 0.003).

### 3.4. The Relationship Between Fatigue and Pain Interference, Pain Related Distress, and Fear of Pain: Hypotheses 2 and 3 (H2a–c and H3a–c)

**Figure 2:**
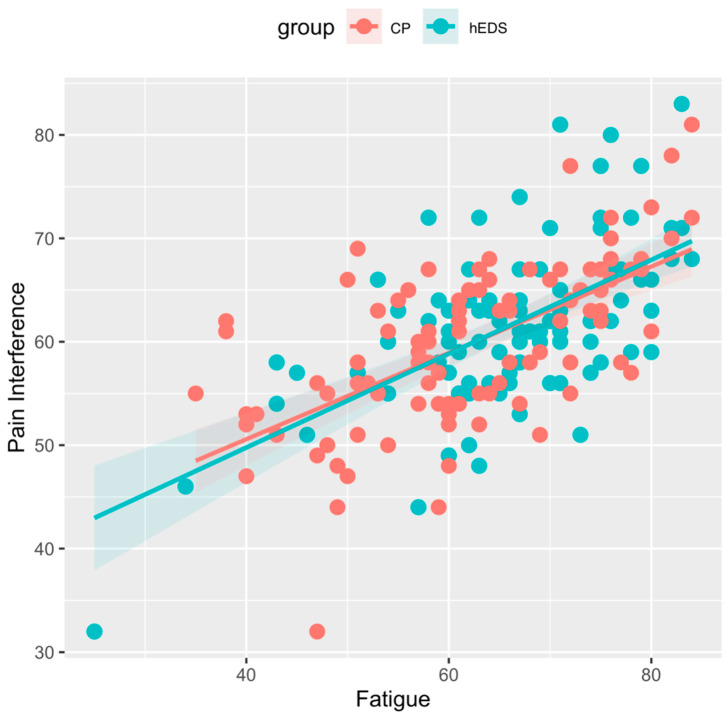
Association of pain interference and fatigue in hEDS and CP groups. Legend: hEDS = group with hypermobile Ehlers–Danlos syndrome; CP = group with chronic pain and no diagnosed hypermobility; Fatigue = PROMIS pediatric fatigue scores; Pain Interference = PROMIS pediatric pain interference scores.

**Figure 3:**
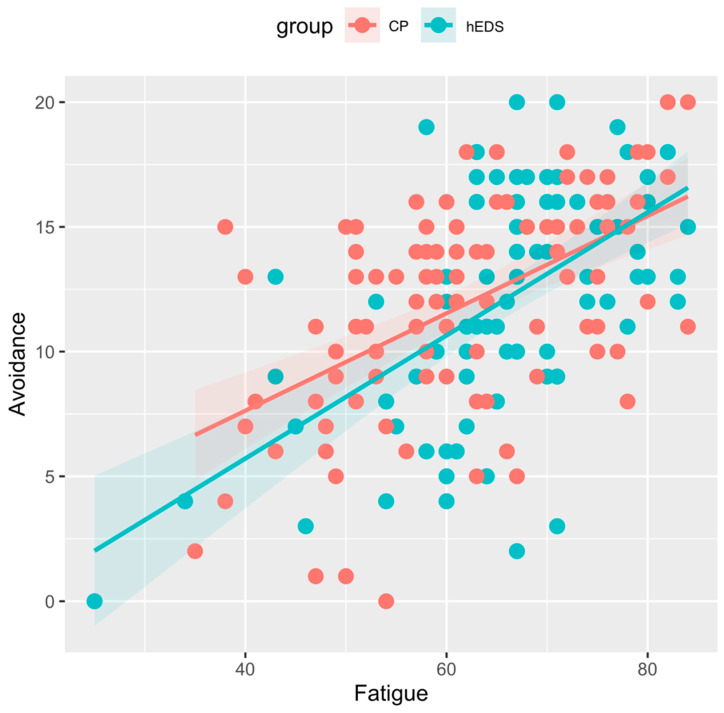
Association of avoidance and fatigue in hEDS and CP groups. Legend: hEDS = group with hypermobile Ehlers–Danlos syndrome; CP = group with chronic pain and no diagnosed hypermobility; Fatigue = PROMIS pediatric fatigue scores; Avoidance = Child Fear of Pain Questionnaire—Avoidance of Activities Subscale scores.

**Figure 4:**
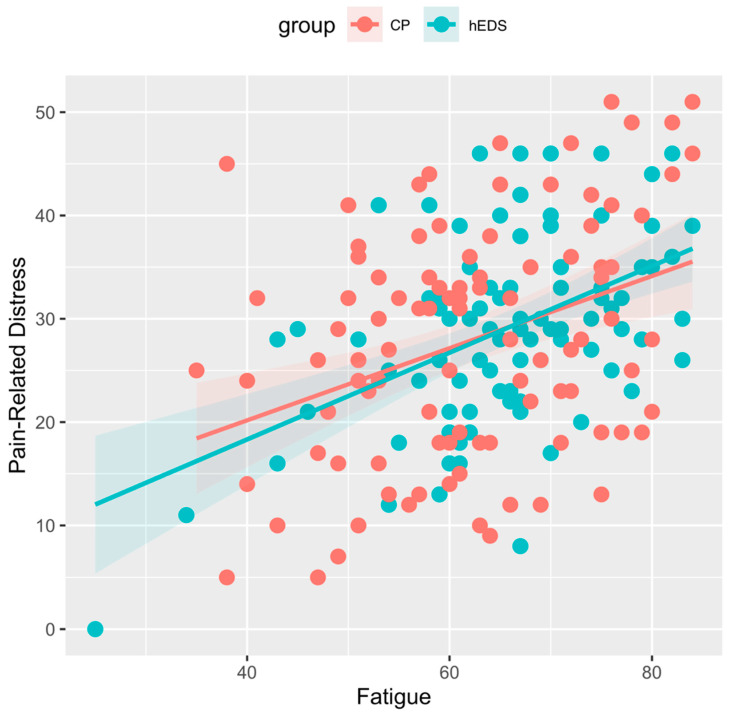
Association of pain-related distress and fatigue in hEDS and CP groups. Legend: hEDS = group with hypermobile Ehlers–Danlos syndrome; CP = group with chronic pain and no diagnosed hypermobility; Fatigue = PROMIS pediatric fatigue scores; Pain-Related Distress = Pain Catastrophizing Scale for Children scores.

As expected, fatigue was significantly related to pain interference (H2a; F(1, 96) = 30.68; *p* < 0.000), avoidance (H2b; F(1, 95) = 30.04; *p* < 0.000), and pain-related distress (H2c; F(1, 96) = 7.29, *p* < 0.001) in those with hEDS while controlling for pain intensity and anxiety. Contrary to our hypotheses, the interaction effect of fatigue and group was not significant for analyses of covariance for pain interference (H3a; F(1, 194) = 0.05; *p* = 0.819), avoidance (H3b; F(1, 192) = 0.97; *p* = 0.326), and pain-related distress (H3c; F(1, 193) = 0.23; *p* = 0.630) while controlling for pain intensity and anxiety across the two groups. See Figure 2, Figure 3 and Figure 4.

## 4. Discussion

Youth with hEDS are at risk for continued pain in adulthood and a poorer quality of life given that hEDS is a lifelong condition and given findings from other pediatric populations demonstrating the impact of early-life chronic pain on poor pain-related adult outcomes [47,48]. Youth with hEDS are also at risk for a poor health-related quality of life and increased disability due to fatigue and deconditioning [26,47] as well as due to under-recognition and stigma surrounding hEDS [11,49]. Fatigue is known to be an important clinical target often understudied but found to be important in its impact on longitudinal outcomes for youth with chronic pain [50]. Youth with hEDS are frequently misdiagnosed or overlooked, and delayed diagnosis is common [24,51,52]. Barriers to diagnosis and care include lack of exposure to patients with EDS and lack of familiarity with diagnostic criteria and common symptoms; pediatric physicians have expressed a desire for treatment and care plan recommendations for youth with EDS [53]. Furthermore, individuals with hEDS experience social and internalized stigma as well as stigma from teachers and healthcare providers in childhood and adulthood [49,54]. Therefore, there is a need to look at variables, such as fatigue, that are clinically meaningful to the experience and treatment of hEDS to offer insights on clinical features and tailored treatment approaches for pediatric hEDS.

The current study’s aims were to (1) determine the prevalence of severity of fatigue in youth with hEDS compared to peers with chronic pain, (2) highlight that fatigue is significantly related to clinically meaningful predictors of outcomes in youth with hEDS such as pain interference, pain-related distress, and avoidance, and (3) understand whether there is a differential relationship between fatigue and the aforementioned factors between groups. Key study findings were higher fatigue severity and prevalence in youth with hEDS compared to peers with chronic pain. Fatigue was significantly related to pain interference, pain-related distress, and activity avoidance in both groups. These relationships did not differ significantly between groups. There could be multiple reasons for the lack of difference between groups, and one explanation may be the overlap between the measure of fatigue utilized (PROMIS fatigue evaluates impairment on emotional and physical function) and items on the pain interference scale (which also assesses functional impairments).

The greater severity and prevalence of clinically elevated fatigue in youth with hEDS compared to peers with other types of chronic pain found in the current study suggest that fatigue may be a factor of particular importance to consider when treating youth with pain and hypermobility. Additionally, youth with hEDS are more vulnerable to deconditioning, which can then exacerbate joint laxity and pain symptoms. Chronic bed rest and exercise deconditioning can also worsen POTS, a condition commonly comorbid with hEDS [55]. Due to these autonomic and musculoskeletal vulnerabilities, it is especially important to address fatigue and associated deconditioning during pain treatment for youth with hEDS. It will be important to also address pain-related distress and activity avoidance as they are associated with fatigue and could impede treatment. In line with the previous literature, this study supports the need for multidisciplinary care for pediatric chronic pain and hypermobility [10,56,57] by demonstrating the relationship between fatigue and pain-related psychological factors. Physical therapy is known to be critical in treating chronic fatigue and pain psychology targets the emotional components of fatigue and its impact on functional outcomes. A recent systematic review [57] supports the co-administration of physical therapy and psychological intervention as beneficial for adolescents with pain and hypermobility, reducing pain interference and pain-related fear [30]. Cognitive behavioral approaches that target pain-related distress and avoidance behaviors may be particularly helpful in the treatment of youth with hEDS in close collaboration or cotreatments with physical therapy for personalized approaches for this population.

Given the results of this study, that fatigue is more severe and more prevalent in youth with hEDS compared to peers with chronic pain, future research may examine how this difference impacts other variables such as mood and social functioning, as well as whether fatigue has a differential impact on chronic pain treatment response in youth with hypermobility. Future studies may focus on examining the differential effect of slow and graded reconditioning on hypermobile and non-hypermobile patients with chronic fatigue, the impact of successful pain treatment on chronic fatigue outcomes, and the presence of chronic fatigue complaints in non-hypermobile family members when compared to patients with hEDS. Future research may also benefit from comparing sleep quality between hypermobile and non-hypermobile youth with chronic pain and investigating the potential differential impact of sleep quality and fatigue on pain-related outcomes between groups. There is a growing yet limited body of literature on fatigue as an important clinical target [50,58] that is often less understood and therefore undertreated in the treatment of chronic pain in children. Its acuity in youth with hEDS makes it even more important to pay attention to fatigue when working with this population.

An important limitation to consider is that, due to the retrospective nature of the study, not all youth in the chronic pain comparison group were explicitly screened for hypermobility. hEDS is frequently misdiagnosed or overlooked, and delayed diagnosis is common [24,52]. As such, it is possible there may be some youth with undiagnosed hypermobility in the comparison group. Additionally, the recent literature suggests that the 2017 diagnostic criteria may not be suitable for diagnosing young children with hEDS and is better suited to adolescents and adults, who may show more symptoms as they mature [59,60]. Future studies may apply updated diagnostic criteria as they continue to evolve. An additional limitation of the study is that power calculations were not run in advance of data collection. Future studies may run a priori power calculations for improved study design. Strengths of this study are a large sample size compared to the existing literature and a matched comparison group structure. This is also the first study comparing groups of youth with chronic pain with and without hEDS.

In the current study, youth with hEDS experienced greater fatigue compared to peers with chronic pain. For all youth in the study, fatigue was positively associated with pain interference, pain-related distress, and avoidance. While the current study did not demonstrate a difference between groups in the strength of the relationship between fatigue and pain-related functioning and psychological variables, the difference in the prevalence of clinically elevated fatigue and fatigue severity was significant. Future studies may explore how this increased fatigue in hypermobile youth contributes to differential longitudinal outcomes. The symptomology and diagnosis of hEDS is often overlooked and misunderstood in youth with chronic pain. Clinicians and researchers should pay close attention in screening for hypermobility in youth with chronic pain and fine-tune treatment approaches to focus on fatigue to improve outcomes in this overlooked and stigmatized group.

## Figures and Tables

**Table 1:** Demographics and baseline characteristics.

	hEDS	CP
Race	-	-
Asian	6%	9%
Black or African American	2%	1%
Other	9%	20%
White or Caucasian	61%	45%
Declines to State	9%	7%
Unknown	13%	18%
Ethnicity	-	-
Hispanic	8%	19%
Not Hispanic	69%	55%
Declines to State	7%	7%
Unknown	16%	19%
Age	14.66 (1.98)	14.67 (1.98)
Legal Sex	-	-
Female	88%	88%
Male	12%	12%
Pain Intensity	6.10 (1.67)	5.88 (2.34)
Anxiety	56.57 (9.83)	54.50 (11.08)

## Data Availability

The deidentified dataset is not publicly available due to Stanford Medicine policy regarding patient privacy and data sharing. Requests to access the dataset should be directed to Dr. Bhandari (rbhandar@stanford.edu) to inquire regarding the possibility of a data-sharing agreement.
